# S100A4 suppresses cancer stem cell proliferation via interaction with the IKK/NF-κB signaling pathway

**DOI:** 10.1186/s12885-018-4563-7

**Published:** 2018-07-25

**Authors:** Yongtong Zhu, Yao Zhou, Xuan Zhou, Yangchun Guo, Daxiong Huang, Jialin Zhang, Chunyan Wang, Longmei Cai

**Affiliations:** 1 Reproductive Medicine Center, Department of Obstetrics and Gynecology, Nanfang Hospital/The First School of Clinical Medicine, Southern Medical University, Guangzhou, 510515 China; 2 Department of Neurology, Integrated Hospital of Traditional Chinese Medicine, Southern Medical University, Guangzhou, 510315 China; 3 Department of Radiation Oncology, Nanfang Hospital, Southern Medical University, Guangzhou, 510515 China

**Keywords:** S100A4, Cancer stem cells, Proliferation, IKK, NF-κB

## Abstract

**Background:**

Bladder cancer often recurs due to incomplete elimination of the cancer stem cells (CSCs). Therefore, new strategies targeting bladder CSCs are needed and the aim of this study was to investigate the effect of S100A4 on the proliferation capacity of MB49 bladder cancer stem cells (MCSCs).

**Methods:**

MCSCs were established and validated. The expression level of S100A4 in MCSCs and MB49 cells was evaluated using Western blotting and quantitative polymerase chain reaction (QPCR). S100A4 was overexpressed or knocked-down by transfection of pCMV6-XL5-S100A4 plasmid or RNA interference (RNAi) respectively. Proliferation capacity of MCSC was evaluated by cell proliferation assay and in vivo tumorigenicity study. Transcriptional activity of nuclear factor kappa B (NF-κB) was analyzed using luciferase reporter assay, and the level of interleukin (IL)-2 as well as tumor necrosis factor (TNF) was quantified by QPCR. Protein-protein interaction of S100A4 and inhibitor of nuclear factor kappa B NF-κB kinase (IKK) was analyzed by immunoprecipitation.

**Results:**

S100A4 was significantly up-regulated in MCSCs, which positively associated with the proliferation capacity, as well as the level of NF-κB, IKK, IL-2 and TNF in MCSCs. Knock-down of S100A4 could reverse such effects. Using immunoprecipitation assay, an interaction between S100A4 and IKK could be observed.

**Conclusions:**

S100A4 is upregulated in MCSCs and possibly enhance the proliferation ability of MCSCs by way of activating the IKK/NF-κB signaling pathway, and S100A4 maybe a hopeful therapeutic target for MCSCs.

## Background

Bladder cancer is a most common urological cancer in China, and the rest of the world [1]. Radical cystectomy with pelvic lymphadenectomy is the standard treatment for bladder cancer. However, bladder cancer may recur due to the incomplete elimination of cancer stem cells (CSCs). Therefore, new strategies targeting bladder CSCs are urgently needed.

*S100A4*, also known as metastasin/FSP1/pEL98/mts-1, is a gene encoding a small calcium binding protein that interacts with other proteins to enhance apoptosis, cell motility, and tumorigenesis [2]. *S100A4* is overexpressed in most cancers, including breast cancer, gastric cancer, and non-small cell lung cancer (NSCLC) [3]. In addition, *S100A4* expression is correlated with patients’ outcome and cancer metastasis [4]. It has been recently reported that S100A4 is a novel marker and a critical regulator of glioma stem cells, with the enhanced S100A4 expression contributing to the presentation of a metastatic phenotype [5]. These findings indicate that S100A4 may be a promising therapeutic target for bladder CSCs. Through bioinformatics analysis in preliminary experiments, we found that there was a close relationship between S100A4 protein and the nuclear factor kappa B (NF-κB) signaling pathway.

In the present study, we demonstrate that *S100A4* is up-regulated in MB49 bladder cancer stem cells (MCSCs). Additionally, overexpression of *S100A4* enhances the proliferation capacity of MCSCs in vitro, and also upregulates inhibitor of nuclear factor kappa B NF-κB kinase (IKK) and activates the NF-κBsignaling pathway, whereas knockdown of *S100A4* resulted in the opposite effects. The findings of this study suggest that S100A4 may promote the proliferation capacity and upregulate IKK in MCSCs by activating the NF-κB signaling pathway. Therefore, S100A4 may have the potential to be a therapeutic target in MCSCs.

## Methods

### Establishment and characterizations of MCSCs

MCSCs were obtained from MB49 bladder cancer cells, which was a mouse cell line, using limited dilution and serum-free culture medium method described previously [6]. The serum-free culture medium was consisted of RPMI1640 supplemented with leukemia inhibitory factor (20 ng/ml, eBioscience, San Diego, CA), basic fibroblast growth factor (20 ng/ml, Peprotech, Rocky Hill, NJ), epidermal growth factor (20 ng/ml, Peprotech), bovine serum albumin (4 μg/ml, Thermo Scientific HyClone, Logan, UT), and B-27 serum-free supplement (20 μl/ml, Invitrogen, Grand Island, NY).

The validation of MCSCs was performed as previously reported [6]. Cancer stem cell markers CD133 and CD44 was detected by flow cytometry analysis, Western blotting, and quantitative polymerase chain reaction (QPCR). The proliferative ability and susceptibility to chemotherapy were examined by Cell Counting Kit-8 reagent assay. Cell migration ability was examined with the transwell assay. The tumorigenic ability was verified using nude mice.

### Western blot analysis

The MB49 cells and MCSCs were respectively harvested. Equal amount proteins were extracted from cells, and separated by 10% sodium dodecyl sulfate -polyacrylamide gel electrophoresis followed by transferring to polyvinylidene difluoride membranes (Millipore, Billerica, MA). The membranes were blocked with 5% skim milk in PBS, and incubated overnight at 4 °C with the primary antibodies including anti-S100A4 (Abcam, Cambridge, MA), anti- IKK (Abcam) and anti-β-actin antibody (Abcam) followed by secondary antibodies (Abcam). Bands were visualized using Fluor Chem FC2 (Alpha Innotech, San Leandro, CA).

### Quantitative polymerase chain reaction

Total RNA was extracted using the Arcturus PicoPure RNA isolation kit (Applied Biosciences, Carlsbad, NM). RNA quality was tested using the Bioanalyzer RNA Pico Chip (Agilent Technologies, Santa Clara, CA). Total RNA were transcribed reversely with Superscript III (Invitrogen), followed by synthesizing the first-strand cDNA which was amplified using a SYBR green PCR master mix (Bio-Rad, Hercules, CA) performed on a 7500 real-time PCR system (AB Applied Biosystems, Singapore). The cycling systems were denaturation (95 °C for 10 s), annealing and extension (60 °C for 60 s). The primers were designed using Primer Express version 2.0 (Applied Biosystems, Foster City, CA), and are shown in Table 1. The relative expression level were analyzed using the ∆∆C_t_ method. *GAPDH* was used as the internal control.

**Table 1:** Primers of selected genes

Gene name	Primers (forward/reverse)	Base pairs of product
S100A4	F: 5’- CCCTGGATGTGATGGTGT-3’	615 bp
	R: 5’- GTTGTCCCTGTTGCTGTC-3’	
Interleukin (IL)-2	F: 5’- GAATGGAATTAATAATTACAAGAA-3’	401 bp
	R; 5’-TGTTTCAGATCCCTTTAGTTCCAG-3’	
Tumor necrosis factor (TNF)	F: 5’- CCAGGCAGTCAGATCATCTTCTC-3’	179 bp
	R: 5’- AGCTGGTTATCTCTCAGCTCCAC-3’	
GAPDH	F: 5’-CCATGGAGAAGGCTGGGG-3’	198 bp
	R: 5’-CAAAGTTGTCATCCATGAC-3’	

### Plasmid construction, and RNA interference and transfection

Full length of *S100A4* gene were inserted into a vector plasmid pCMV6-XL5 (OriGene Technologies). MCSC cells were transfected with pCMV6-XL5-S100A4 plasmid (pCMV-S100A4) performing in Lipofectamine™ 2000 (Invitrogen) following the manufacturer’s instructions, which were referred to as MCSCs/S100A4-vector. The expression of *S100A4* was detected at 48 and 72 h after transfection on transcription and translation level respectively.

*S100A4* was knockdown by S100A4-siRNA transfection. Double-stranded siRNAs specific to *S100A4* were bought from Shanghai GenePharma Co., Ltd. (Shanghai, China). The *S100A4* siRNA sequences were 5’-TGTAACGAATTCTTTGAAG-3′, and 5’-ACGAATTCTTTGAAGGCTT-3′. The non-coding (NC) siRNAs sequence was 5’-UUCUCCGAACGUGUCACGUTT-3′. MCSCs were transfected with a final concentration of 20 nM of siRNA performing in Lipofectamine™ 2000, which were referred to as MCSCs/S100A4-siRNA or MCSCs/NC-siRNA cells, respectively.

### Cell counting Kit-8 (CCK-8) assay

Cells were plated at a density of 1 × 10^3^ cells per well in a 96-well plate, followed by incubating for 72 h. After incubation, cell counting kit-8 reagent (CCK-8, Dojindo Molecular Technologies, Kumamoto, Japan) was added to each well with 10 μl at a time periods of 24, 48, and 72 h. Incubated for 4 h, the absorbance value was read at 450 nm performing in EnSpire 2300 multilabel reader (PerkinElmer, Singapore).

### In vivo tumorigenicity study

All animal experiments were obeyed the Chinese animal protection laws and guidelines, and approved by the Ethics Committee of Southern Medical University (Contract 1,116,904).

Four-week-old immune deficient nude mice were purchased from Experimental Animals Center (Southern Medical University, Guangzhou, China), and fed under specific, pathogen-free conditions. Cells (1 × 10^4^) were injected into mice subcutaneously. Tumor xenograft formation was recorded at 10, 20, 30, and 45 days, calculated the tumor volume according to the formula d^2^ × D/2, where D and d were the longest and the shortest diameters, respectively. Then mice were sacrificed after CO_2_ anesthesia.

### Luciferase reporter assay

NF-κB transcriptional activity was examined using the pNF-κB-luciferase reporter and control plasmids (Clontech, Mountain View, CA). The cells were plated at a sub confluent density, followed by co-transfecting with 0.5 μg of NF-κB luciferase reporter plasmid or negative plasmid, and 0.02 μg of Renilla luciferase pRL-TK plasmid (Promega, Madison, WI) performing in Lipofectamine 2000 reagent (Invitrogen). Cell lysates were prepared 24 h after transfection, and the reporter activity was measured using the Dual-luciferase reporter assay system (Promega).

### Immunoprecipitation

Cells were washed with ice-cold PBS, followed by lysing in Tris-buffered saline (pH 7.4), containing 150 mM NaCl, 50 mM Tris, 0.1% Nonidet P-40, 1 mM EDTA, 1 mM Na_3_VO_4_, 10 mM NaF, 2.5 mg/ml aprotinin and leupeptin, 1 mM β-glycerophosphate and 4-(2-aminoethyl) benzenesulfonyl fluoride hydrochloride, and 10 mM iodoacetate. After incubation, cellular debris and nuclei were removed by centrifugation. Cell lysates were incubated with specific antibody overnight, and then with Protein A-Sepharose (Amersham Biosciences, Piscataway, NJ) beads for another 4 h. The immunoprecipitates were washed in Tris-buffered saline four times and boiled in Laemmli buffer included of 0.02% blue bromophenol and 2% bmercaptoethanol.

### Statistical analysis

SPSS19.0 software was used for all statistical analyses. Numeric data were described as the mean value ± standard deviation. Comparisons were performed by Students t-test. A value of *P* < 0.05 was considered to indicate statistical significance.

## Results

### Expression of S100A4 in MCSC MB49 cells

**Fig. 1:**
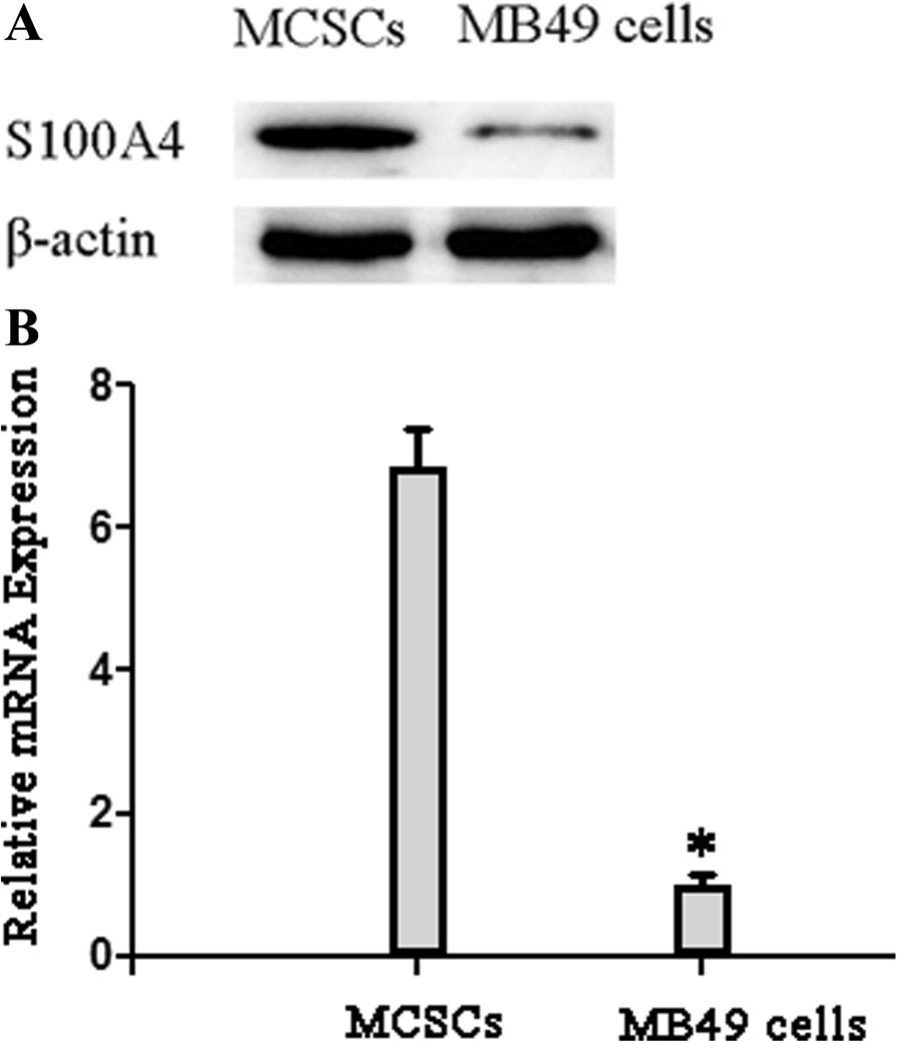
*S100A4* was upregulated in MCSCs. **a** Western blotting analysis of S100A4 protein expression in MCSCs and MB49 cells; β-actin was used as a loading control. **b** Quantitative PCR of *S100A4* mRNA expression in MCSCs and MB49 cells. Transcript levels were normalized to GAPDH, and expressed relative to MB49 cells. Data is mean ± SD of three independent experiments. *P < 0.05

S100A4 level in MCSCs was increased as detected by Western blotting (Fig. 1a), and *S100A4* mRNA expression level in MCSCs was also significantly increased as detected by QPCR (Fig. 1b).

### Effects of transfection on MCSCs

**Fig. 2:**
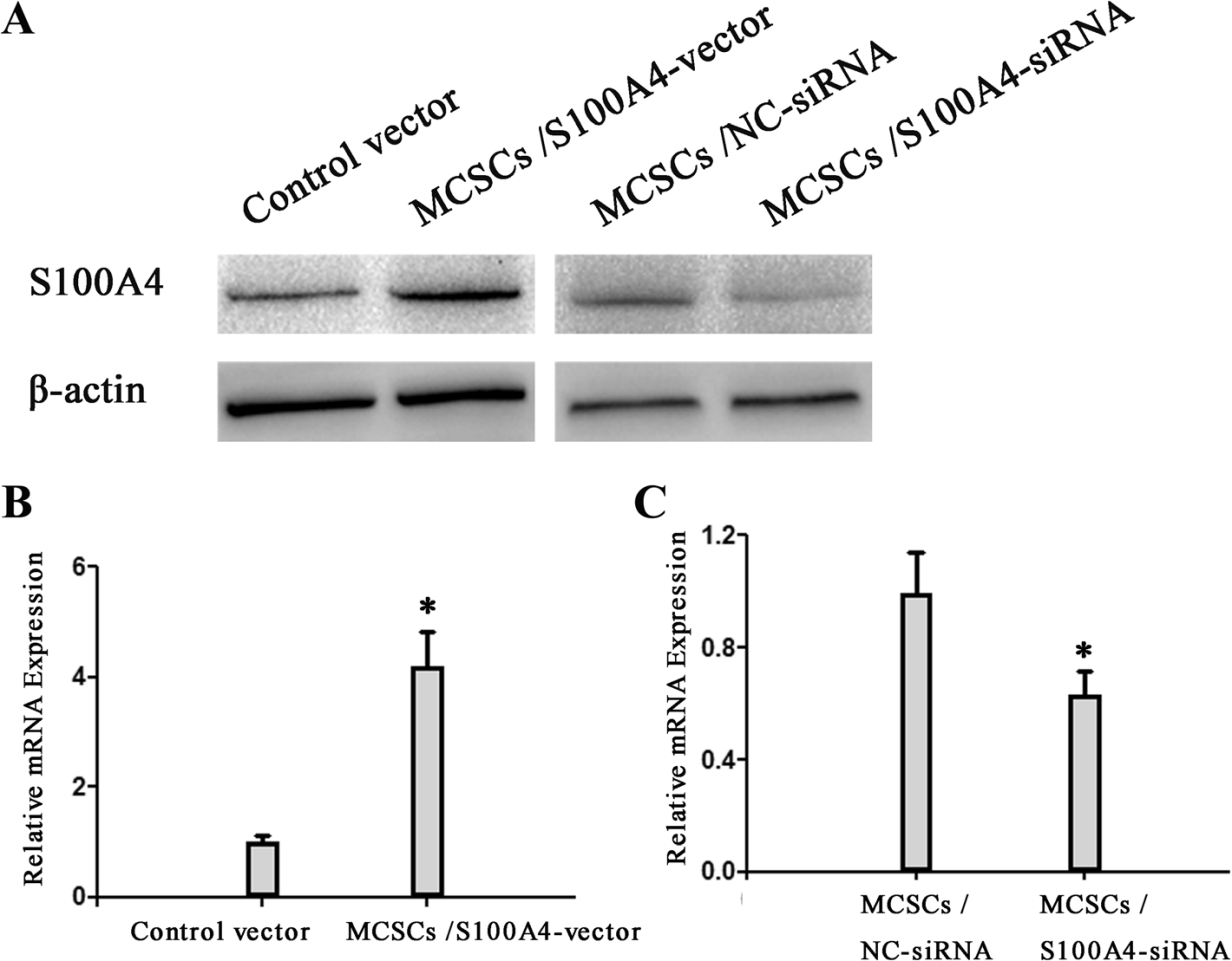
Effects of transfection on MCSCs. **a** *S100A4* protein level of was detected by Western blotting. **b** The mRNA expression of *S100A4* in MCSCs transfected with S100A4 vector was determined by quantitative PCR. **c** The mRNA expression of *S100A4* in MCSCs transfected with *S100A4*-siRNA was determined by quantitative PCR. Data is mean ± SD of three independent experiments. *P < 0.05

MCSCs were transfected with *S100A4*-vector or *S100A4*-siRNA, and the transfection efficiency was examined in vitro. As shown in Fig. 2a, protein level of *S100A4* was increased in *S100A4*-vector group, while which was inhibited in *S100A4*-siRNA group. *S100A4* mRNA expression was increased by transfecting *S100A4*-vector (Fig. 2b, *P* < 0.05), while suppressed by *S100A4*-siRNA transfection (Fig. 2c, *P* < 0.05).

### Effects of S100A4 on MCSC proliferation

**Fig. 3:**
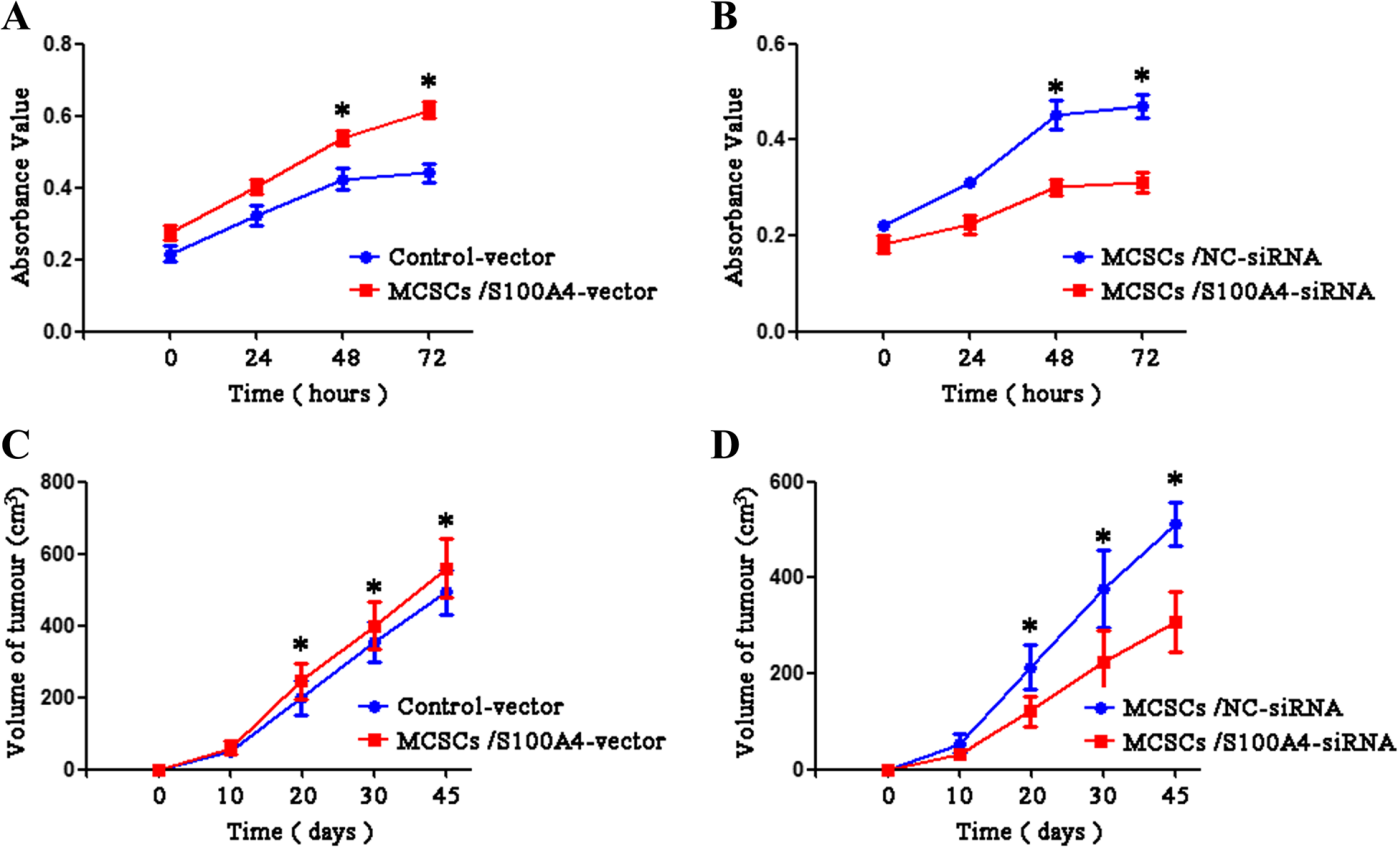
*S100A4* enhanced the proliferation ability of MCSCs. **a** The cell proliferation growth curve using the Cell Counting Kit (CCK)-8 assay showed that MCSCs transfected with *S100A4*-vector exhibit a higher absorbance value. **b** The CCK-8 assay showed that MCSCs transfected with *S100A4*-siRNA exhibited a lower absorbance value. **c** MCSCs transfected with S100A4-vector caused an increased tumor volume. **d** MCSCs transfected with S100A4-siRNA caused a decreased tumor volume. Data is mean ± SD of three independent experiments. *P < 0.05

The proliferation capacity of transfected MCSCs was evaluated by Cell Counting Kit-8 and tumorigenicity assay. The cell proliferation curve was increased by *S100A4* overexpression (Fig. 3a), while was decreased by *S100A4* suppression (Fig. 3b)., Tumor volume was increased by *S100A4* overexpression, while was decreased by *S100A4* suppression (Fig. 3c and d, respectively).

### S100A4 promotes the activity of NF-κB and the transcription of its target genes

**Fig. 4:**
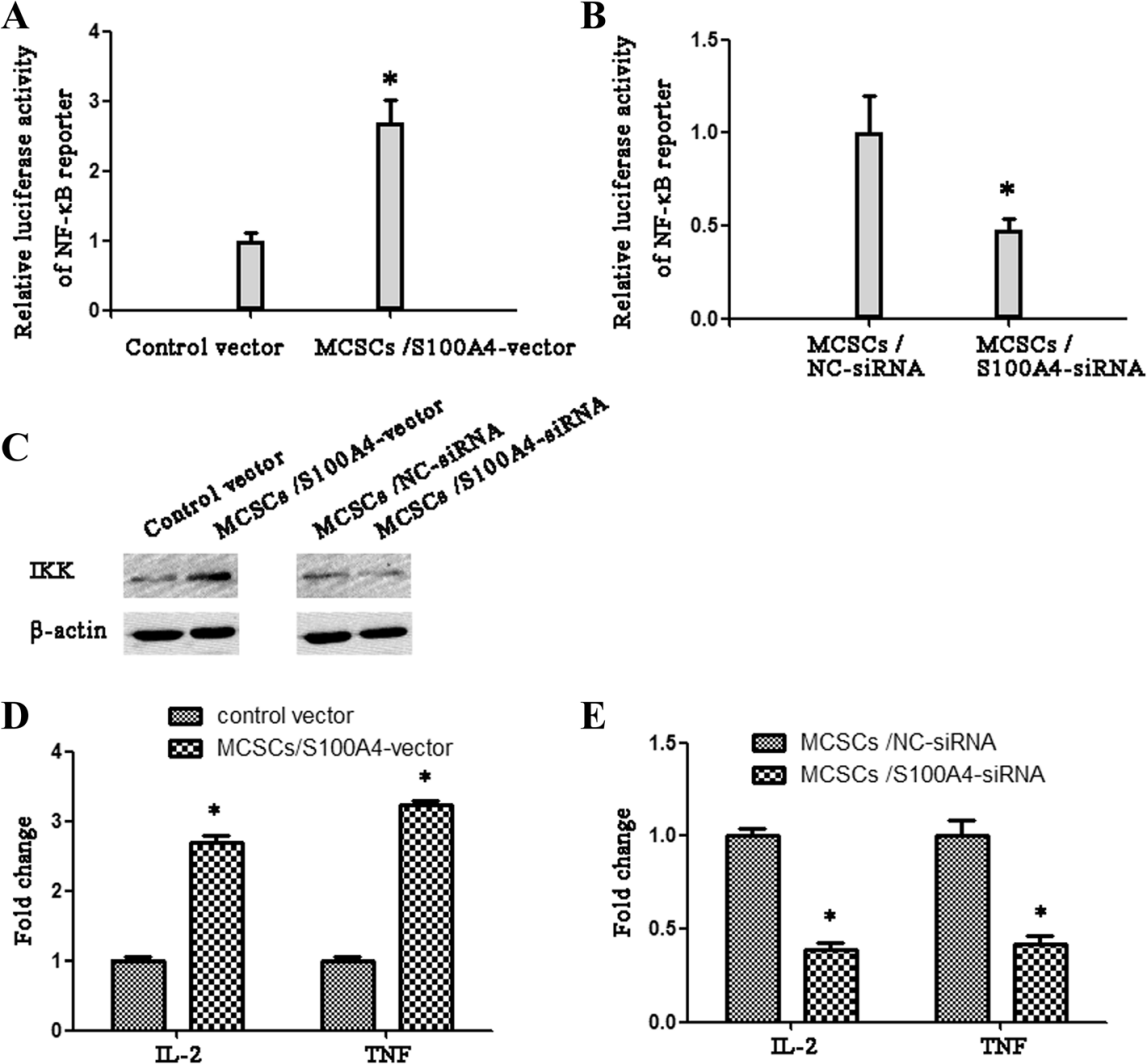
*S100A4* promoted NF-κB transcriptional activity. **a** Luciferase reporter assay of NF-κB transcriptional activity showed that MCSCs transfected with *S100A4*-vector exhibited enhanced transcriptional activity of a NF-κB reporter gene. **b** Luciferase reporter assay showed that MCSCs transfected with *S100A4*-siRNA exhibited decreased NF-κB transcriptional activity. **c** Western blotting analysis of the expression of IKK; β-actin was used as a negative control. **d** Quantitative PCR analysis showed that the expression of IL-2 and TNF were up-regulated in MCSCs transfected with *S100A4*-vector. **e** Quantitative PCR analysis showed that the expression of IL-2 and TNF were down-regulated in MCSCs transfected with *S100A4*-siRNA. Data is mean ± SD of three independent experiments. * *P* < 0.05

Overexpression of *S100A4* enhanced the transcriptional activity of a NF-κB reporter gene (Fig. 4a), which was suppressed when knockdown of *S100A4* (Fig. 4b). Western blotting indicated that overexpression of *S100A4* increased the level of IKK (Fig. 4c). In addition, several NF-κB target genes, including IL-2 and TNF, were up-regulated in *S100A4*-overexpressing cells (Fig. 4d), and downregulated in *S100A4*-silenced MCSCs (Fig. 4e). Taken together, these results indicated that the NF-κB pathway may participate in the proliferation effect of *S100A4* in MCSCs.

### S100A4 regulates NF-κB activation through IKK

**Fig. 5:**
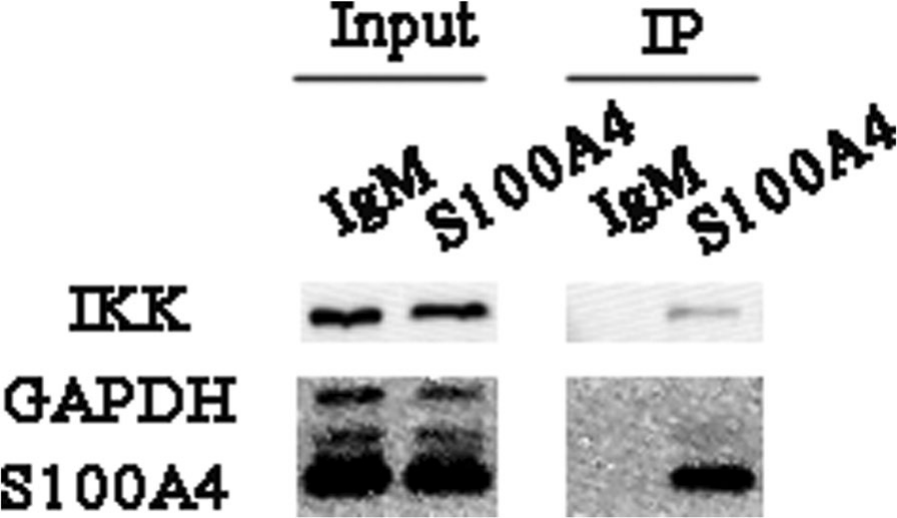
S100A4 interacted with IKK. MCSCs extracts were immunoprecipitated with mouse IgM or anti-S100A4 antibody, and then subjected to immunoblot analysis of the IKK protein

We hypothesized that S100A4 may regulate NF-κB signaling pathway through a direct interaction with IKK. To verify this hypothesis, interaction between S100A4 and IKK were analyzed using immunoprecipitation assays. As shown in Fig. 5, S100A4 physically interacted with IKK.

## Discussion

S100A4 has been reported to be an important regulator for modulating the cell cycle, proliferation, apoptosis, and migration in different kinds of cells through various mechanisms [7]. In adult animals, S100A4 expression is restricted to very few kinds of normal tissue or cells, whereas it is usually overexpressed in cancerous tissues [2]. However, little is known about the function of S100A4 on bladder CSCs. In this research, we demonstrate firstly that S100A4 is able to enhance the proliferation capacity of mouse bladder MCSCs. Another study has indicated that S100A4 could be a novel marker and regulator of glioma stem cells in human and murine malignant gliomas [5]. Different expression levels of S100A4 lead to different stem cell characteristics: it promotes self-renewal at a lower level, while promotes quiescence through asymmetric stem progenitor divisions at a higher level [5]. Several works have also demonstrated that *S100A4* may have oncogenic effects in a multitude of tumor types [8]. However, further researches are needed to manifest the exact function of S100A4 in various cancer stem cells.

In the present research, we found that cell proliferation was significantly prompted by *S100A4* overexpression, while was inhibited by *S100A4* suppression. Luciferase reporter assays manifested that the transcriptional activity of NF-κB was enhanced significantly by overexpression of *S100A4*, implying NF-κB may play an crucial part in the S100A4-induced proliferation capacity of MCSCs.

NF-κB is the collective name of a family of transcription factors consisting of seven proteins, encoded by five genes: c-Rel, RelA, RelB, p100/p52 and p105/p50 [9]. NF-κB has been widely known for its regulatory effects on immunological and inflammatory processes, like a serious of other pathological and physiological responses containing of the development and progression of cancer. NF-κB activation is mediated frequently by plenty of chemotherapeutic agents, which generally means inducing a strong anti-apoptotic response which limits the efficacy of treatments [10]. As shown in this research, activation of NF-κB signaling is regulated by IKK in a negative way. The IκB family of inhibitory proteins generally holds the NF-κB pathway in an inactive status by sequestering NF-κB in the cytoplasm. Many extracellular stimuli could result in the activation of IKK [11]. Following stimulation, IKK is recruited to the combined signaling complex of late around membrane receptors, which affording a platform in phosphorylation and activation subsequently [12]. As many works have reported, the accurate regulation of IKK activity is an important procedure in activating NF-κB pathway [13]. Accordingly, the regulation of IKK recruitment is crucial in activating NF-κB induced by an extracellular stimulation.

Consistent with those previous works, our research also demonstrated that overexpression of S100A4 upregulated the level of IKK, followed by raising the activation of NF-κB ultimately. In addition, overexpression of S100A4 upregulated some genes, IL-2 and TNF, which downstream of the NF-κB signaling pathway. IL-2 has been demonstrate to play an crucial role in tumor proliferation [14]. TNF-α takes a paramount role in proliferation during the development and progression in different kinds of cancer [15]. Accordingly, it would be attractive to detect whether IL-2 or TNF act a part in proliferation and disease progression in MCSCs.

There are some limitations to this study that needed to be taken into account. The IKK complex is formed by three subunits: IKKα, IKKβ, and IKKγ [16], and it would be better to test the individual subunits separately rather than IKK alone. In addition, our study did not test whether S100A4 enhanced the stemness of MB49 cells or not, and it would be better to overexpressed or knocked-down S100A4 not only in MCSCs but also in MB49 cells.

## Conclusion

In summary, the present work showed that S100A4 is upregulated in MCSCs and possibly enhance the proliferation ability of MCSCs by way of activating the IKK/NF-κB signaling pathway. These results may offer a mechanisms for regulation of proliferation in MCSCs, and S100A4 maybe a hopeful therapeutic target for MCSCs.

## Abbreviations

CCK-8: Cell counting kit-8
CSCs: Cancer stem cells
IKK: Inhibitor of NF-κB kinase
IL-2: Interleukin-2
MCSCs: MB49 bladder cancer stem cells
NF-κB: Nuclear factor kappa B
QPCR: Quantitative polymerase chain reaction
TNF: Tumor necrosis factor
